# Dilution susceptibility testing method evaluation for the combination of ceftibuten and avibactam against Enterobacterales

**DOI:** 10.1128/jcm.00072-25

**Published:** 2025-10-21

**Authors:** O. N. Walser, J. Howland, K. Jankowski, A. Kennedy-Mendez, G. G. Stone, C. M. Pillar, D. A. Hufnagel

**Affiliations:** 1 Microbiologics Antibiotic and Microbiome Research Center 827187 , Kalamazoo, Michigan, USA; 2 Pfizer 105623 , Groton, Connecticut, USA; Mayo Clinic, Baltimore, Maryland, USA

**Keywords:** ESBL, antibiotic resistance, susceptibility testing, clinical methods, Enterobacterales, preclinical drug studies, beta-lactams

## Abstract

With the increase in antimicrobial resistance and multidrug-resistant infections, the discovery of additional therapeutic options to combat different infection types and treat different patient populations is paramount. Many recently developed antibiotics are administered intravenously, which can needlessly overburden healthcare systems and may not be needed or appropriate for every patient. Extended-spectrum beta-lactamase (ESBL)-producing Enterobacterales infections have increased since 2012 and are resistant to first-line therapies used for many infections including complicated urinary tract infections. To address the need for alternatives to intravenous therapies against ESBL-producing Enterobacterales, a novel combination of ceftibuten and a modified prodrug version of avibactam (ARX-1796) designed for oral administration is being developed to treat serious Gram-negative infections. Because clinicians often prescribe antibiotics to patients based on the susceptibility profile of the bacterium causing their infection—particularly when empiric therapy fails—establishing the parameters for the susceptibility testing is a vital phase of development. To address two key gaps in reference method development for CTB/AVI, this study follows CLSI M23 Tier 1 guidance to investigate the impact of nonstandard testing conditions on broth microdilution testing, as well as the correlation between broth microdilution and agar dilution methods. There was a high correlation between the methods for CTB/AVI (essential agreement ≥90%) when evaluating 153 Enterobacterales isolates, including recent clinical isolates with diverse antimicrobial resistance mechanisms. Besides 100-fold increased inoculum density and pH of 5.0, CTB/AVI activity was largely unaffected by 21 alternate broth microdilution test conditions.

**IMPORTANCE:**

New antibiotics are desperately needed to combat expanding and new antimicrobial resistance trends globally. While there are antibiotics in the drug development pipeline that target antibiotic-resistant bacteria, most of these new antibiotics are not available in an oral formulation. Ceftibuten in combination with avibactam targets drug-resistant ram-negative organisms, including those that cause complicated urinary tract infections, and is orally administered. This combination fills a gap for clinicians seeking an appropriate oral therapeutic regimen for patients with drug-resistant infections. During anti-infective development, it is important to delineate the variables for susceptibility testing so that clinicians can confidently evaluate whether an infecting organism is resistant or susceptible to a potential therapy. This study evaluated dilution susceptibility testing methods for ceftibuten in combination with avibactam against targeted organisms (Enterobacterales) and found that broth and agar dilution testing methods agree, and this combination is recalcitrant to most variations, aside from low pH and inoculum size, in standard test parameters.

## INTRODUCTION

The rise of antimicrobial-resistant (AMR) infections and the burden related to the intravenous (IV) therapies administered to treat them underscore the need for continued development of novel oral antimicrobials. The most recent forecasts estimate that 8.22 million AMR-associated deaths could occur by the year 2050 and that continued development of new drugs targeting drug-resistant Gram-negative isolates could avert the deaths of 11.1 million people over the next 25 years (1). The novel antimicrobial development pipeline is weighted toward IV therapies, with less than a third (12/40) of the antimicrobials in the 2023 pipeline designed for oral administration (2). When patients with serious drug-resistant infections require IV therapies, they are administered either during an inpatient visit or on an outpatient basis at home and administered by a caregiver, at a clinic, or at a hospital, which can increase the burden on the healthcare system and the patient (3, 4). Due to the burdens associated with IV therapy, the timing of switching patients from IV to oral therapy is an evolving area of research, with several studies suggesting that earlier transitions to oral therapy once a patient is past the critical period result in similar clinical outcomes as longer durations of IV therapy (5). Therefore, efforts to expand the suite of novel antimicrobials that are orally available have the potential to lessen the impact of serious infections on patients and healthcare systems.

Avibactam (AVI) is a novel diazabicyclooctane beta-lactamase inhibitor that targets extended-spectrum beta-lactamases (ESBL) including class A, class C, and some class D serine-beta-lactamases (e.g., TEM/SHV, CTX-M, AmpC, KPC, and OXA) (6). The Centers for Disease Control and Prevention (CDC) Antibiotic-Resistant Threats Report lists ESBL-producing Enterobacteriaceae as a serious threat that has been increasing in frequency since 2012 (7). AVI was approved for IV administration with ceftazidime (CAZ/AVI) in 2015, initially for urinary tract and intra-abdominal infections caused by Enterobacterales and *P. aeruginosa* (8). Additionally, an aztreonam (ATM)/AVI combination was recently developed to address the need for the combined activity of ATM and AVI to target isolates with metallo-beta lactamases (MBL) for use against Enterobacterales and other serious Gram-negative infections (9, 10). These newest beta-lactam/AVI combinations address the need for novel therapeutics against serious Gram-negative infections, but they do not fulfill the need for novel oral therapies.

The only oral beta-lactam/beta-lactamase inhibitor available is the combination amoxicillin-clavulanate, which is active against Enterobacterales isolates carrying ESBLs *in vitro*, but clinical use against these organisms is associated with treatment failure (11). Ceftibuten (CTB) is a third-generation cephalosporin that is recalcitrant to hydrolysis by many ESBLs, and a combination with AVI (CTB/AVI) is being developed to target serious Gram-negative infections (12–14). The formulation under development is particularly distinct because it combines with the novel AVI prodrug, ARX-1796, and is designed for oral administration (13–15). ARX-1796 addresses both the need to target beta-lactamase-producing Gram-negative bacteria and the need for oral formulations (15).

As a part of advancing CTB/AVI to the next phase of drug development, testing parameters need to be evaluated (16). The parameters for testing are established using Tier 1 studies such as the equivalency between broth versus agar MIC values and the impact of nonstandard test conditions (medium pH, surfactant, inoculum densities, etc.) on the perceived activity of the test agent following guidance from CLSI M23 (16). These types of studies ensure that clinical laboratories—which may vary in their antimicrobial susceptibility testing practices—are equipped to evaluate the antimicrobial susceptibility of investigational antimicrobials such as CTB/AVI (16) and that the standard method provides robust reproducible results. Automated systems used in most clinical laboratories must be cleared by the United States Food and Drug Administration (FDA), so multi-year delays between FDA approval of a new antimicrobial and their incorporation into an FDA-cleared automated susceptibility testing system are typical (17). Recent changes have simplified the process through which the FDA recognizes breakpoints established by the CLSI in an effort to reduce the time laboratories must wait for devices to be cleared for use, but FDA funding cuts are expected to complicate this transition (17). Thus, the availability of comprehensive reference testing parameters remains relevant to patient care.

Despite the fact that the oral formulation of CTB/AVI under development uses the prodrug ARX-1796, antimicrobial susceptibility testing must be conducted with AVI rather than ARX-1796 (13, 14). Testing with AVI at a fixed combination of 4 µg/mL was established previously (14, 16). Additionally, the setting of quality control (QC) ranges is required prior to use of investigational breakpoints during clinical trials, where antimicrobial susceptibility testing is conducted on isolates from trial participants (16). These QC ranges for CTB/AVI have already been established by broth microdilution and were provisionally published in the 33rd edition of CLSI M100 in 2023, accompanied by a statement regarding the lack of data on the equivalency of broth MIC values with those determined by agar dilution (13, 18) that is still in the 35th edition (2025) (19). In this study, we evaluated the equivalency between MIC values of CTB/AVI determined by agar dilution relative to broth microdilution, as well as the impact of nonstandard test conditions on CTB/AVI MIC values.

## MATERIALS AND METHODS

### Test compounds

AVI was provided by Pfizer (Lot No. PF-06416494-02 (0002 E1)) and solubilized in water or directly in the test medium. CTB was either provided by Pfizer (Lot No. 6701570001E1) or obtained from Sigma (Billerica, MA; Catalog No. 116512; Lot No. 124697) and solubilized in dimethyl sulfoxide (DMSO). While the CLSI-recommended solvent/diluent for ceftibuten is now 0.1 mol/L phosphate buffer (pH 8.0)/water or 0.1 mol/L phosphate buffer (pH 8.0) (19), DMSO is often utilized for both solvent/diluent during preclinical development. In fact, the solvent/diluent for ceftibuten used to be 10% DMSO/water (20). As seen below, CTB/AVI was consistently found to be in the acceptable QC range. Meropenem (MEM) was obtained from the United States Pharmacopeia (USP; Rockville, MD; Catalog No. 1392454; Lot No. J0K434) and solubilized in water. MIC values for MEM, CTB, and AVI alone can be found in Table S1.

### Test organisms

The test organisms evaluated in this study consisted of nonduplicate, nonconsecutive clinical isolates from the Microbiologics Repository (MMX; Kalamazoo, MI) and reference isolates from the American Type Culture Collection (ATCC; Manassas, VA), the National Collection of Type Cultures (NCTC; Salisbury, UK), and the CDC (Atlanta, GA). A total of 153 isolates were evaluated, including those selected to account for many different resistance phenotypes, including but not limited to various beta-lactamases (BL) and MBL. Table S2 provides BL content information on the isolates where available.

### Broth microdilution MIC assay (standard format)

MIC values were determined using a broth microdilution procedure described by the CLSI (19, 21). The test medium used was cation-adjusted Mueller Hinton broth (CAMHB; BD, Sparks, MD; Lot Nos. 1242967, 1089790), with AVI added to a final test concentration of 4 µg/mL when testing CTB/AVI. Isolates were streaked from frozen stocks onto trypticase soy agar (TSA) with 5% sheep blood and were incubated at 35°C overnight in preparation for testing. Media preparation, liquid handling, incubation, and panel reading were all performed as previously described (22).

### Broth microdilution MIC assay (non-standard conditions)

Nonstandard test parameters were evaluated in parallel with standard parameters during the study as described below:

#### pH

The effect of altered CAMHB test medium pH was assessed at pH 5.0, 6.0, 7.4 (reference pH), and 8.0.

#### Inoculum Effect

An initial 0.5 McFarland cell suspension (approximately 1–2 × 10^8^ CFU/mL) was prepared and diluted to achieve final inoculum concentrations targeting 5 × 10^4^, 5 × 10^5^ (reference inoculum), 5 × 10^6^, and 5 × 10^7^ CFU/mL in the assay. Cell densities were determined by serial 10-fold dilution and track-dilution plating 10 µL onto TSA. Plates were angled to allow for the cell suspension to track down the agar surface. After cell suspensions had dried on the agar surface, plates were inverted and incubated at 35°C overnight, prior to enumeration of colony-forming units (CFUs).

#### Atmosphere of Incubation

Assays were conducted in ambient atmosphere (standard atmosphere) and in the presence of approximately 5% CO_2_ using standard media.

#### Cation Supplementation

The effect of cations was determined by assaying the compounds in unadjusted Mueller-Hinton Broth (MHB; BD, Sparks, MD; Lot No. 9156832) supplemented to 5 mg/L Ca^2+^ and 5 mg/L Mg^2+^, 25 mg/L Ca^2+^ and 5 mg/L Mg^2+^, 5 mg/L Ca^2+^ and 12.5 mg/L Mg^2+^, 50 mg/L Ca^2+^ and 25 mg/L Mg^2+^, as well as MHB supplemented to the standard 25 mg/L Ca^2+^ and 12.5 mg/L Mg^2+^ (reference cation supplementation). MHB was supplemented to result in the final Ca^2+^ and Mg^2+^ concentrations listed above by using a 10 mg/mL stock of MgCl_2_ and 10 mg/mL stock of CaCl_2_, each of which was prepared in accordance with CLSI guidelines (19, 21).

#### Incubation Time

Standard incubation time for the evaluated organisms is 16 to 20 hr. The effect of prolonged incubation time on MIC values was determined by reading MIC values following incubation for 18 hr (standard), 24 hr, and 48 hr in standard test media.

#### Human Serum and Serum Albumin Supplementation

Assays were conducted in standard media in the presence and absence of pooled human serum and human serum albumin. Filter-sterilized pooled human serum (Innovative Research, Novi, MI; Cat. No. ISERABHI100ML; Lot No. 36199) was added at a final concentration of 10% and 50% (vol/vol) to CAMHB. Human serum albumin (Millipore Sigma; Billerica, MA; Lot No. 2935512) was added at a final concentration of 4% (wt/vol) to CAMHB and filter-sterilized before use in the assay.

#### Urine

The impact of incubation in pooled normal human urine (Innovative Research; Novi, MI; Cat. No. ISERABHI100ML; Lot No. 32739) and pooled normal urine adjusted to neutral pH (7.2–7.4) was evaluated when compared to CAMHB at pH 7.2–7.4, as well as CAMHB adjusted to the pH of the pooled normal human urine (pH 6.8, as measured upon receipt). All solutions were filter-sterilized prior to use in the assay.

#### Media Supplements

The impact of testing in media containing 3% lysed horse blood (LHB; Hemostat; Dixon, CA; Lot No. 652081) and media containing 0.002% polysorbate-80 (P-80; Alfa Aesar; Ward Hill, MA; Cat. No. L13315; Lot No. 10197649) were evaluated relative to the standard medium.

### Agar dilution MIC assay

When MIC values were determined using the agar dilution method, all the serial dilution and liquid handling steps were performed manually using pipettes. CTB was prepared and diluted for agar dilution according to CLSI guidelines (19, 21).

CTB was mixed with molten (50°C to 55°C) Mueller Hinton agar (MHA; BD; Cat. No. 212322; Lot No. 1242967) with and without 4 µg/mL of AVI (final concentration in the assay). CTB was added at a ratio of 0.3 mL 100X test agent to 29.3 mL agar. Once test agents were added to the agar in a sterile tube, they were mixed gently and then poured into a sterile square petri plate (100 × 100 mm). Plates were allowed to solidify at room temperature and placed in a laminar air flow hood with the covers off to remove condensed moisture on the agar surface.

Using the same inoculum used for broth microdilution MIC testing (the 1:10 diluted 0.5 McFarland suspension described above), each bacterial cell suspension was then transferred to wells in a stainless-steel replicator block for agar dilution evaluation across 6 separate run days.

Each agar plate containing either drug or no drug (control) was stamped with a stainless-steel Steer’s replicator. The prongs on the replicator deliver approximately 1–2 µL of inoculum to an agar surface. The resulting inoculum spots contained approximately 10^4^ cells. Inoculated plates were placed on the bench top with the agar surface face up to allow for the inoculum to soak into the agar. The plates were inverted and incubated at 35°C for 20 hr. The MIC was defined as the lowest test agent concentration that completely inhibited bacterial growth on the agar surface (21). Agar and broth MIC values were compared, and essential agreement was determined by grouping the percent of isolates that had a broth MIC within 2-fold of the agar MIC.

## RESULTS

### Impact of alternative conditions on broth microdilution testing

CTB, AVI, and CTB/AVI were evaluated with a total of 21 alternate test conditions including media pH, inoculum size, atmosphere of incubation, incubation time, divalent cation concentrations, and the presence of additional supplements (human serum, human serum albumin, urine, blood, and surfactant) on three isolates of *Escherichia coli* and three isolates of *Klebsiella pneumoniae*. MEM evaluation was from 0.004 to 4 µg/mL, below the QC range for *K. pneumoniae* ATCC-BAA-1705 and preventing interpretations for alternate conditions for this isolate and MEM. *Pseudomonas aeruginosa* ATCC 27853 was also included in the study as a non-Enterobacterales QC organism for MEM.

#### Medium pH

There was a trend toward decreased activity of CTB/AVI when tested in media at low pH (5.0 and 6.0) relative to standard conditions across isolates Table 1, and this was most apparent at pH 5.0 with isolates of *K. pneumoniae*. The activity of CTB alone and AVI alone was also decreased at low pH Table S1. For MEM, MIC values were within 4-fold of the standard conditions at low pH for most organisms except when testing against *E. coli* ATCC 25922 where the MIC was elevated 8-fold at pH 5.0 Table S1.

**TABLE 1:** MIC values of ceftibuten in combination with avibactam when tested under standard and nonstandard conditions by broth microdilution

Conditions	MIC (µg/mL) | Ceftibuten-avibactam						
Isolate	*E. coli*			*K. pneumoniae*			*P. aeruginosa*
	ATCC 25922	NCTC 13353	ATCC BAA-2523	ATCC BAA-1705	ATCC 700603	CDC 0504	ATCC 27853
pH 5.0	0.25/4^*b*^	0.12/4	0.25/4	2/4^*b*^	2/4^*b*^	8/4	>8/4
pH 6.0	0.12/4	0.12/4	0.12/4	0.25/4	0.25/4	0.5/4	>8/4
pH 7.4^*a*^	0.06/4 (0.016/4–0.12/4)	0.06/4 (0.03/4–0.12/4)	0.06/4	0.12/4 (0.03/4–0.25/4)	0.12/4 (0.06/4–0.25/4)	0.06/4	>8/4
pH 8.0	0.06/4	0.12/4	0.06/4	0.12/4	0.12/4	0.06/4	>8/4
Inoculum ~10^4^ CFU/mL	0.06/4	0.06/4	0.06/4	0.12/4	0.12/4	0.06/4	>8/4
Inoculum ~10^5^ CFU/mL	0.06/4 (0.016/4–0.12/4)	0.12/4 (0.03/4–0.12/4)	0.06/4	0.12/4 (0.03/4–0.25/4)	0.12/4 (0.06/4–0.25/4)	0.12/4	>8/4
Inoculum ~10^6^ CFU/mL	0.06/4	0.12/4	0.12/4	0.25/4	0.25/4	0.12/4	>8/4
Inoculum ~10^7^ CFU/mL	>8/4^*b*^	>8/4^*b*^	>8/4	>8/4^*b*^	>8/4^*b*^	>8/4	>8/4
Ambient atmosphere	0.03/4 (0.016/4–0.12/4)	0.06/4 (0.03/4–0.12/4)	0.06/4	0.12/4 (0.03/4–0.25/4)	0.12/4 (0.06/4–0.25/4)	0.12/4	>8/4
5% CO_2_ atmosphere	0.06/4	0.06/4	0.06/4	0.25/4	0.12/4	0.12/4	>8/4
MHB w/ 25 Ca^2+^/ 12.5 Mg^2+^ (mg/L)	0.06/4 (0.016/4–0.12/4)	0.06/4 (0.03/4–0.12/4)	0.06/4	0.12/4 (0.03/4–0.25/4)	0.25/4 (0.06/4–0.25/4)	0.12/4	>8/4
MHB w/ 5 Ca^2+^/ 5 Mg^2+^ (mg/L)	0.06/4	0.12/4	0.06/4	0.12/4	0.25/4	0.12/4	>8/4
MHB w/ 25 Ca^2+^/ 5 Mg^2+^ (mg/L)	0.12/4	0.12/4	0.03/4	0.12/4	0.12/4	0.12/4	>8/4
MHB w/ 5 Ca^2+^/ 12.5 Mg^2+^ (mg/L)	0.06/4	0.12/4	0.12/4	0.12/4	0.25/4	0.12/4	>8/4
MHB w/ 50 Ca^2+^/ 25 Mg^2+^ (mg/L)	0.06/4	0.06/4	0.06/4	0.12/4	0.25/4	0.06/4	>8/4
18 hr incubation	0.03/4 (0.016/4–0.12/4)	0.06/4 (0.03/4–0.12/4)	0.06/4	0.12/4 (0.03/4–0.25/4)	0.12/4 (0.06/4–0.25/4)	0.12/4	>8/4
24 hr incubation	0.06/4	0.06/4	0.06/4	0.12/4	0.12/4	0.12/4	>8/4
48 hr Incubation	0.06/4	0.06/4	0.06/4	0.12/4	0.12/4	0.12/4	>8/4
No human serum/albumin	0.06/4 (0.016/4–0.12/4)	0.06/4 (0.03/4–0.12/4)	0.12/4	0.12/4 (0.03/4–0.25/4)	0.12/4 (0.06/4–0.25/4)	0.12/4	>8/4
10% human serum	0.12/4	0.06/4	0.12/4	0.12/4	0.12/4	0.12/4	>8/4
50% human serum	0.06/4	0.06/4	0.12/4	0.12/4	0.25/4	0.25/4	>8/4
4% human serum albumin	0.12/4	0.25/4^*b*^	0.25/4	0.25/4	0.5/4^*b*^	0.5/4	>8/4
Urine (pH 7.0)	0.016/4	0.12/4	0.06/4	0.03/4	0.03/4^*b*^	0.06/4	>8/4
Urine (pH 7.4)	0.016/4	0.06/4	0.03/4	0.03/4	0.06/4	0.03/4	>8/4
CAMHB (pH 7.4)	0.06/4 (0.016/4–0.12/4)	0.12/4 (0.03/4–0.12/4)	0.06/4	0.12/4 (0.03/4–0.25/4)	0.12/4 (0.06/4–0.25/4)	0.06/4	>8/4
CAMHB (pH 7.0)	0.06/4	0.06/4	0.06/4	0.12/4	0.25/4	0.06/4	>8/4
3% lysed horse blood	0.06/4	0.06/4	0.06/4	0.12/4	0.25/4	0.12/4	>8/4
0.002% P-80	0.06/4	0.25/4^*b*^	0.06/4	0.12/4	0.25/4	0.06/4	>8/4
No supplementation	0.06/4 (0.016/4–0.12/4)	0.06/4 (0.03/4–0.12/4)	0.06/4	0.12/4 (0.03/4–0.25/4)	0.12/4 (0.06/4–0.25/4)	0.06/4	>8/4

^*a*^ Cells shaded gray represent MIC values as observed under standard conditions. CLSI QC ranges shown in parentheses where applicable.

^*b*^ MIC value fell outside of the CLSI acceptable QC range in a condition for the indicated isolate.

#### Inoculum size

At increased inoculum densities (approximately 10^7^ CFU/mL), MIC values were elevated relative to standard inoculum density for CTB/AVI, CTB alone, and AVI alone against all evaluated *E. coli* and *K. pneumoniae* isolates Table 1; Table S1. An increase in MEM MIC values was also observed at this highest inoculum density when tested against all isolates where MEM was active Table S1.

#### Atmosphere of incubation and incubation time

There were no notable impacts of incubation in the presence of CO_2_ on the activity of CTB/AVI, CTB, AVI, or MEM against nearly all evaluated organisms Table 1; Table S1. Exceptions include slight decreases in activity of CTB alone and MEM against *E. coli* ATCC BAA-2523 and AVI against *K. pneumoniae* ATCC BAA-1705 in an atmosphere of 5% CO_2_ Table S1.

The activities of CTB/AVI and MEM were unaltered by extended incubation times Table 1. AVI alone exhibited increased MIC values during prolonged incubation for two of the evaluated *E. coli* Table S1, but not for remaining isolates. CTB alone was unaffected by the incubation length for all organisms except for *E. coli* ATCC BAA-2523, where MIC values increased during prolonged incubation Table S1.

#### Altered divalent cation concentrations

CTB/AVI, CTB alone, AVI alone, and MEM activity was largely not affected when tested in media with nonstandard cation concentrations relative to standard concentrations against all tested organisms, with the majority of reads within a doubling dilution of standard conditions with varied cation concentrations against all evaluated isolates Table 1; Table S1.

#### Human serum/albumin, urine, surfactant, or blood

There was a trend toward 2-fold to 4-fold higher CTB/AVI MIC values in the presence of 4% human serum albumin Table 1. Similar results were apparent with CTB alone and MEM where these agents were active Table S1. There was no notable impact of testing in the presence of 10% or 50% human serum on the activity of CTB/AVI, with equivalent MIC values in serum or within 2-fold of those observed in the standard medium Table 1. There were select instances where AVI alone was less active in 50% serum, particularly with the *E. coli* isolates where 8-fold increases in the MIC for AVI alone were observed Table S1.

CTB/AVI and CTB testing in urine generally led to lower MIC values at both pH 7 and 7.4 Table 1. AVI and MEM MIC values were within 4-fold of the standard method in all instances, except where AVI alone MIC values were 16-fold lower in urine when testing against *K. pneumoniae* ATCC 700603 Table S1.

CTB/AVI, CTB, and AVI activities were mostly unaffected by the presence of P-80 or 3% lysed horse blood across all evaluated organisms with singular exceptions Table 1; Table S1. When testing with the addition of P-80 to the test media, CTB/AVI MIC values increased 4-fold when testing against *E. coli* NCTC 13353 Table 1, while AVI alone had a >16-fold increase in the MIC when testing against *E. coli* ATCC 25922 and a 4-fold decrease in the MIC when testing against *K. pneumoniae* ATCC 700603 Table S1.

To summarize, a total of 21 alternate test conditions were evaluated against each isolate. Against the four isolates with CTB/AVI QC ranges, there were 2, 3, 2, and 4 instances of the CTB/AVI MIC values falling outside of the QC range when testing in alternate conditions against *E. coli* ATCC 25922, *E. coli* NCTC 13353, *K. pneumoniae* ATCC BAA-1705, and *K. pneumoniae* ATCC 700603, respectively. That means that even in alternate conditions, CTB/AVI was still in QC in 90.5%, 85.7%, or 81.0% of the evaluations.

### Assessment of CTB/AVI by broth microdilution and agar dilution

**Fig 1:**
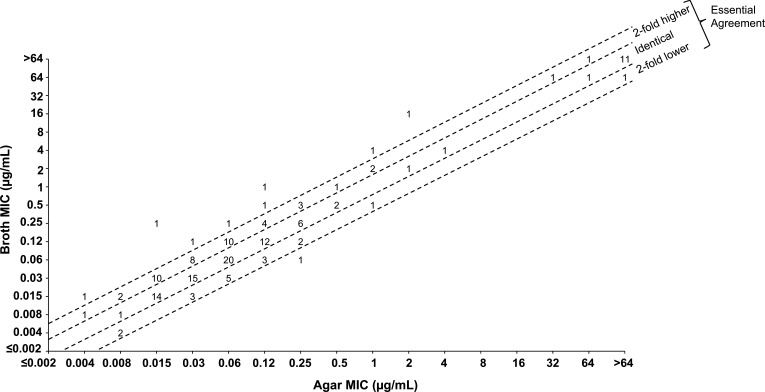
Scatterplot comparing ceftibuten/avibactam broth and agar MIC values against Enterobacterales (*N* = 153). MIC values displayed represent the ceftibuten concentration of the combination, where avibactam was tested at a fixed concentration of 4 µg/mL by both methods.

Essential agreement between the methods was defined as having MIC values by both methods that were either identical or within 2-fold of each other. As shown in Table 2 and Fig. 1, there was good agreement between the two testing methods when evaluating CTB/AVI against isolates of Enterobacterales, with 93.6% essential agreement overall, and >93% essential agreement by species, with the exception of the “other Enterobacterales” (including *Serratia* spp., *Morganella* spp., and *Providencia* spp.) where 89.5% essential agreement was observed. Where >4-fold differences in MIC values were observed between methods, there was a trend toward higher MIC values when testing in broth rather than by agar dilution. No obvious trends toward discordance were observed with regards to species or resistance mechanism for CTB/AVI. The 93.6% overall essential agreement satisfies the CLSI M23 guidance of ≥90% essential agreement for establishing the equivalency of the reference testing methods. Additionally, 20 replicates of the four QC organisms (*E. coli* ATCC 25922, *E. coli* NCTC 13353, *K. pneumoniae* ATCC BAA-1705, and *K. pneumoniae* ATCC 700603) were also evaluated by broth microdilution and agar dilution with concurrent inocula. All 80 agar dilution tests were within the acceptable broth microdilution QC range Table S3.

**TABLE 2:** Comparison of MIC values for ceftibuten/avibactam and ceftibuten alone as determined concurrently by broth microdilution and agar dilution susceptibility testing against Enterobacterales

Ceftibuten/avibactam									
Organism	N^*a*^	EA^*b*^	Fold-difference—broth MIC vs agar MIC						
			≥–8	–4	–2	0	+2	+4	≥+8
Enterobacterales	140	93.6%		1	16	73	42	5	3
*E. coli*	33	93.9%			2	19	10	1	1
*Klebsiella* spp.	34	94.1%		1	6	19	7	1	
*Proteus* spp.	19	94.7%			2	9	7		1
*E. cloacae*	16	93.8%			1	7	7	1	
*Citrobacter* spp.	19	94.7%			3	10	5	1	
Other^*c*^	19	89.5%			2	9	6	1	1

Ceftibuten									
Enterobacterales	132	80.3%		3	8	53	45	16	7
*E. coli*	32	81.3%			1	13	12	5	1
*Klebsiella* spp.	33	90.9%		1	2	15	13	2	
*Proteus* spp.	19	57.9%				7	4	4	4
*E. cloacae*	15	86.7%		1		6	7		1
*Citrobacter* spp.	16	87.5%		1	3	6	5	1	
Other	17	70.6%			2	6	4	4	1

^*a*^ Number of evaluable isolates (isolates for which the fold-difference as indicated could be determined based on the MIC); isolates with undefined MIC values (e.g., >64 µg/mL) are not included in this analysis but are shown in scatterplots (Fig. 1 and 2).

^*b*^ EA: essential agreement (MIC value identical or within 2-fold between methods; shaded in gray).

^*c*^ Other Enterobacterales consisted of four *Serratia marcescens*, nine *Morganella morganii*, and six *Providencia* spp.

**Fig 2:**
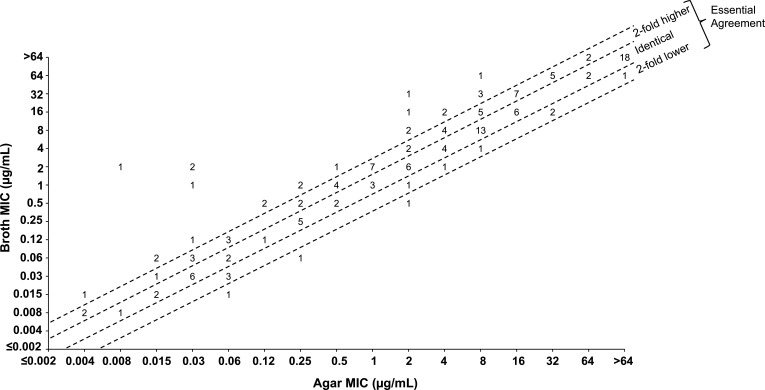
Scatterplot comparing ceftibuten broth and agar MIC values against Enterobacterales (*N* = 153).

Agreement between MIC values for broth microdilution and agar dilution was lower for CTB alone compared to the CTB/AVI combination, as shown in Table 2; Fig. 1 and 2. Essential agreement was 80.3% between the two testing methods for Enterobacterales overall and ranged from 70% to 90% for most of the tested species, except for *Proteus* spp., where 57.9% essential agreement was observed. Of note, there was heavy trailing observed when testing *Proteus* spp. by broth that was not apparent by agar. There were five instances where CTB MIC values observed with BMD and AD methods differed ≥16-fold, and these can be attributed to four isolates of *Proteus* spp. and one *Morganella morganii* isolate Table S2. As with the CTB/AVI combination, the MIC differences of >4-fold were driven by broth MIC values being higher than agar dilution MIC values. Among the six *E. coli* isolates that accounted for CTB discordance, five of these isolates carry a TEM (Class A BL that cleaves AVI). No other obvious trends toward discordance were observed with regards to species or phenotypes.

## DISCUSSION

In this study, among the tested nonstandard test parameters, CTB/AVI MIC values were impacted only by medium pH and inoculum density, and agar dilution and broth microdilution MIC values were largely equivalent when CTB/AVI was tested against Enterobacterales. Isolates for this study were chosen to include a broad range of resistance phenotypes, particularly those carrying BL, to better evaluate the combination and the inhibitory activity of AVI. The MIC values of CTB/AVI decreased in comparison to those of CTB or AVI alone against BL-positive isolates Table 1; Table S1 confirming the anticipated synergy between CTB and AVI in combination against these BL-positive isolates.

The activity of CTB/AVI was negatively impacted by low media pH and increased inoculum size across the evaluated organisms Table 1. For the organisms where QC ranges were established by a prior study (13), the CTB/AVI MIC values shifted outside of the established QC ranges in media with pH 5.0 and in test panels with high inoculum. While a medium pH of 6.0 generally increased MIC values, the QC organisms were still within the CLSI-established acceptable MIC range (19). The decrease in activity in the presence of higher inoculum is not surprising as the inoculum effect is a known phenomenon with beta-lactams (23).

Compared to low pH and high inoculum size, the activity of CTB/AVI was less affected by other alterations made to standard testing parameters in this study including atmosphere of incubation, incubation time, changes to divalent cation concentrations in media, and increased media pH Table 1. For the four isolates with CTB/AVI QC ranges, there were only 2 to 4 alternate test conditions out of 21 total that caused CTB/AVI MIC values to fall out of the QC range. When human serum, surfactant, or blood was present in the testing medium or where panels contained urine in place of media, there were select cases where the impacts to the activity of CTB/AVI were slight, with 4-fold differences in the activity between standard and nonstandard test conditions.

Essential agreement between broth microdilution and agar dilution methods for susceptibility testing of CTB/AVI was high overall against the panel of Enterobacterales isolates (93.6%; Table 2). Most of the CTB/AVI broth microdilution and agar dilution MIC values against this population enriched with BL-positive isolates were at or within a doubling dilution of each other, with the same MIC value being observed with both methods for over half of the tested isolates Table S1. The heavy trailing with CTB alone against *Proteus* spp. accounts for the high level of discordance observed with CTB alone; neither trailing nor discordance was observed when evaluating broth and agar methods for CTB/AVI, which was the primary focus of this study Table 2; Table S1.

With the rise in AMR and the threat of ESBLs, novel therapies for different infection types are becoming more important. According to the CDC Emerging Infections Program, only a third of ESBL-producing Enterobacterales cases in 2021 required hospitalization (24), showing a need for an oral antibiotic to combat the majority of these cases. Expanding the availability of oral therapeutic options for ESBL-producing Enterobacterales infections, including complicated urinary tract infections, will give clinicians a new therapeutic option to address the growing problem of these types of infections. This study provides transparency on the performance of broth microdilution testing for CTB/AVI by showing the impact of alternate test conditions on activity and shows that agar dilution is a suitable susceptibility testing method for this combination. This information is a critical resource for laboratories seeking to test new antimicrobials that have not been incorporated into automated antimicrobial susceptibility testing systems. Overall, CTB/AVI was tested robustly in this study with strong correlation between broth and agar methods, and alterations to the standard broth conditions largely had little effect on MIC values.

## SUPPLEMENTAL MATERIAL

Tables S1, S2, and S3 jcm.00072-25-s0001.pdf MIC values of meropenem, ceftibuten, and avibactam when tested under standard and non-standard conditions against 7 organisms; line listing of broth and agar MIC values (μg/mL) and broth:agar MIC ratios for ceftibuten alone and ceftibuten/avibactam; and line listings of broth and agar MIC values (μg/mL) for ceftibuten/avibactam against QC isolates.

ASM does not own the copyrights to Supplemental Material that may be linked to, or accessed through, an article. The authors have granted ASM a non-exclusive, world-wide license to publish the Supplemental Material files. Please contact the corresponding author directly for reuse.
