# Supplementary material for: Dilution susceptibility testing method evaluation for the combination of ceftibuten and avibactam against Enterobacterales
Source: J Clin Microbiol. 2025 Oct 21;63(11):e00072-25. doi: 10.1128/jcm.00072-25 (PMC12607882; doi:10.1128/jcm.00072-25)
Supplement: Tables S1, S2, and S3 — MIC values of meropenem, ceftibuten, and avibactam when tested under standard and non-standard conditions against 7 organisms; line listing of broth and agar MIC values (μg/mL) and broth:agar MIC ratios for ceftibuten alone and ceftibuten/avibactam; and line listings of broth and agar MIC values (μg/mL) for ceftibuten/avibactam against QC isolates. [file jcm.00072-25-s0001.pdf]

Table S1. MIC values of ceftibuten, meropenem, and avibactam when tested under standard and non-standard conditions by broth microdilution

**Conditions**	**MIC (µg/mL) | Ceftibuten**						
**Isolate**	***E. coli***			***K. pneumoniae***			***P. aeruginosa***
	**ATCC 25922**	**NCTC 13353**	**ATCC BAA-2523**	**ATCC BAA-1705**	**ATCC 700603**	**CDC 0504**	**ATCC 27853**
pH 5.0	0.5	>64[#b]	16	>64[#]	2	>64	64
pH 6.0	0.5	64	8	8	1	64	>64
pH 7.4[a]	0.25 (0.12-0.5)	64 (16-64)	4	16 (4-32)	1	16	64
pH 8.0	1[#]	16	4	16	0.5	4	>64
Inoculum ~10^4 CFU/mL	0.25	32	4	8	0.5	8	64
Inoculum ~10^5 CFU/mL	0.25 (0.12-0.5)	64 (16-64)	4	16 (4-32)	1	32	64
Inoculum ~10^6 CFU/mL	0.5	>64[#]	16	>64[#]	2	>64	>64
Inoculum ~10^7 CFU/mL	>64[#]	>64[#]	>64	>64[#]	>64	>64	>64
ambient atmosphere	0.5 (0.12-0.5)	32 (16-64)	2	8 (4-32)	0.5	16	64
5% CO_2 atmosphere	0.25	64	8	8	1	32	64
MHB w/ 25 Ca^{2+}/ 12.5 Mg^{2+} (mg/L)	0.5 (0.12-0.5)	32 (16-64)	4	16 (4-32)	0.5	32	>64
MHB w/ 5 Ca^{2+}/ 5 Mg^{2+} (mg/L)	0.5	>64[#]	4	16	1	16	>64
MHB w/ 25 Ca^{2+}/ 5 Mg^{2+} (mg/L)	0.5	32	8	16	1	16	>64
MHB w/ 5 Ca^{2+}/ 12.5 Mg^{2+} (mg/L)	0.5	64	8	16	2	32	>64
MHB w/ 50 Ca^{2+}/ 25 Mg^{2+} (mg/L)	0.25	64	4	16	1	8	>64

Conditions	MIC (µg/mL) | Ceftibuten						
Isolate	*E. coli*			*K. pneumoniae*			*P. aeruginosa*
	**ATCC 25922**	**NCTC 13353**	**ATCC BAA-2523**	**ATCC BAA-1705**	**ATCC 700603**	**CDC 0504**	**ATCC 27853**
18 hr incubation	0.5 (0.12-0.5)	32 (16-64)	2	8 (4-32)	0.5	16	64
24 hr Incubation	0.5	32	4	8	0.5	16	64
48 hr Incubation	0.5	32	16	16	0.5	16	>64
no human serum/albumin	0.5 (0.12-0.5)	32 (16-64)	8	16 (4-32)	0.5	32	>64
10% human serum	0.5	32	4	16	1	32	64
50% human serum	0.25	32	4	8	1	16	>64
4% human serum albumin	0.5	>64[#]	16	32	2	64	>64
Urine (pH 7.0)	0.25	32	1	4	0.25	4	16
Urine (pH 7.4)	0.12	16	1	8	0.5	8	64
CAMHB (pH 7.4)	0.5 (0.12-0.5)	32 (16-64)	4	16 (4-32)	0.5	16	64
CAMHB (pH 7.0)	0.25	64	4	8	1	32	64
3% lysed horse blood	0.5	32	4	8	1	8	64
0.002% P-80	0.25	32	4	16	0.5	8	64
no supplementation	0.25 (0.12-0.5)	64 (16-64)	4	16 (4-32)	1	16	64

[a]Cells shaded grey represent MIC values as observed under standard conditions. CLSI QC ranges shown in parentheses where applicable.

[b]#, denotes where an MIC value fell outside of the CLSI acceptable QC range in a condition for the indicated isolate.

Conditions	MIC (μg/mL) | Meropenem						
Isolate	*E. coli*			*K. pneumoniae*			*P. aeruginosa*
	ATCC 25922	NCTC 13353	ATCC BAA-2523	ATCC BAA-1705	ATCC 700603	CDC 0504	ATCC 27853
pH 6.0	0.06	0.03	0.12	>4	0.03	2	0.25
pH 7.4[a]	0.016 (0.008-0.06)	0.03	0.25	>4 (8-64)	0.03	>4	0.25 (0.12-1)
pH 8.0	0.016	0.03	0.5	>4	0.03	>4	1
Inoculum ~10^4 CFU/mL	0.016	0.03	0.12	4[#]	0.016	1	0.25
Inoculum ~10^5 CFU/mL	0.016 (0.008-0.06)	0.03	0.25	>4 (8-64)	0.03	4	1 (0.12-1)
Inoculum ~10^6 CFU/mL	0.03	0.06	1	>4	0.12	>4	1
Inoculum ~10^7 CFU/mL	4[#]	2	4	>4	0.5	>4	>4[#]
ambient atmosphere	0.016 (0.008-0.06)	0.03	0.25	>4 (8-64)	0.03	>4	0.5 (0.12-1)
5% CO_2 atmosphere	0.016	0.03	1	>4	0.03	>4	0.25
MHB w/ 25 Ca^{2+}/ 12.5 Mg^{2+} (mg/L)	0.016 (0.008-0.06)	0.016	0.25	>4 (8-64)	0.016	1	1 (0.12-1)
MHB w/ 5 Ca^{2+}/ 5 Mg^{2+} (mg/L)	0.03	0.03	0.12	>4	0.03	4	0.25
MHB w/ 25 Ca^{2+}/ 5 Mg^{2+} (mg/L)	0.016	0.016	0.25	>4	0.016	1	0.5
MHB w/ 5 Ca^{2+}/ 12.5 Mg^{2+} (mg/L)	0.016	0.03	0.12	>4	0.06	1	0.5
MHB w/ 50 Ca^{2+}/ 25 Mg^{2+} (mg/L)	0.016	0.016	0.25	>4	0.016	1	0.25
18 hr incubation	0.016 (0.008-0.06)	0.03	0.25	>4 (8-64)	0.03	>4	0.5 (0.12-1)
24 hr Incubation	0.016	0.03	0.25	>4	0.03	>4	0.5
48 hr Incubation	0.03	0.03	0.25	>4	0.03	>4	1

Conditions	MIC (µg/mL) | Meropenem						
Isolate	*E. coli*			*K. pneumoniae*			*P. aeruginosa*
	ATCC 25922	NCTC 13353	ATCC BAA-2523	ATCC BAA-1705	ATCC 700603	CDC 0504	ATCC 27853
no human serum/albumin	0.03 (0.008-0.06)	0.03	0.25	>4 (8-64)	0.03	4	0.5 (0.12-1)
10% human serum	0.016	0.03	0.25	>4	0.03	>4	0.5
50% human serum	0.03	0.03	0.5	>4	0.03	>4	1
4% human serum albumin	0.03	0.03	0.25	>4	0.06	>4	0.5
Urine (pH 7.0)	0.016	0.016	0.5	>4	0.016	>4	0.25
Urine (pH 7.4)	0.016	0.016	0.5	>4	0.03	>4	0.25
CAMHB (pH 7.4)	0.016 (0.008-0.06)	0.03	0.12	>4 (8-64)	0.03	4	0.5 (0.12-1)
CAMHB (pH 7.0)	0.016	0.03	0.25	>4	0.016	4	0.25
3% lysed horse blood	0.016	0.03	0.25	>4	0.03	4	0.5
0.002% P-80	0.03	0.03	0.25	>4	0.03	4	1
no supplementation	0.016 (0.008-0.06)	0.03	0.25	>4 (8-64)	0.03	>4	0.25 (0.12-1)

[a]Cells shaded grey represent MIC values as observed under standard conditions. CLSI QC ranges shown in parentheses where applicable.

[b]#, denotes where an MIC value fell outside of the CLSI acceptable QC range in a condition for the indicated isolate.

Conditions	MIC (µg/mL) | Avibactam						
Isolate	*E. coli*			*K. pneumoniae*			*P. aeruginosa*
	ATCC 25922	NCTC 13353	ATCC BAA-2523	ATCC BAA-1705	ATCC 700603	CDC 0504	ATCC 27853
pH 5.0	>64	64	32	>64	>64	>64	>64
pH 6.0	64	32	16	16	64	32	>64
pH 7.4[a]	8	8	8	16	64	32	>64
pH 8.0	8	8	8	8	8	8	>64
Inoculum ~10^4 CFU/mL	4	8	8	8	8	8	>64
Inoculum ~10^5 CFU/mL	32	8	8	16	16	16	>64
Inoculum ~10^6 CFU/mL	32	16	64	>64	>64	>64	>64
Inoculum ~10^7 CFU/mL	>64	>64	>64	>64	>64	>64	>64
ambient atmosphere	8	8	8	8	32	32	>64
5% CO_2 atmosphere	8	8	8	32	64	64	>64
MHB w/ 25 Ca^{2+}/ 12.5 Mg^{2+} (mg/L)	8	8	8	32	>64	16	>64
MHB w/ 5 Ca^{2+}/ 5 Mg^{2+} (mg/L)	8	8	8	16	64	8	>64
MHB w/ 25 Ca^{2+}/ 5 Mg^{2+} (mg/L)	8	8	8	16	>64	16	>64
MHB w/ 5 Ca^{2+}/ 12.5 Mg^{2+} (mg/L)	16	8	8	8	16	32	>64
MHB w/ 50 Ca^{2+}/ 25 Mg^{2+} (mg/L)	16	8	8	64	32	8	>64
18 hr incubation	8	8	8	8	32	32	>64
24 hr Incubation	8	16	16	16	32	32	>64
48 hr Incubation	64	16	>64	16	32	32	>64
no human serum/albumin	8	8	8	32	64	32	>64

Conditions	MIC (μg/mL) | Avibactam						
Isolate	*E. coli*			*K. pneumoniae*			*P. aeruginosa*
	ATCC 25922	NCTC 13353	ATCC BAA-2523	ATCC BAA-1705	ATCC 700603	CDC 0504	ATCC 27853
10% human serum	16	8	8	32	64	8	>64
50% human serum	64	64	64	16	>64	16	>64
4% human serum albumin	16	16	16	8	>64	64	>64
Urine (pH 7.0)	32	8	8	16	8	8	>64
Urine (pH 7.4)	32	8	8	8	8	8	>64
CAMHB (pH 7.4)	8	8	8	8	>64	16	>64
CAMHB (pH 7.0)	8	8	8	8	64	32	>64
3% lysed horse blood	16	8	8	16	16	16	>64
0.002% P-80	>64[b]	16	8	8	16	32	>64
no supplementation	8	8	8	16	64	32	>64

[a]Cells shaded grey represent MIC values as observed under standard conditions. CLSI QC ranges shown in parentheses where applicable.

[b]While MIC value is reported as >64, diminished non-trailing growth was observed starting at 8 μg/mL.

Table S2. Line listing of broth and agar MIC values (µg/mL) and broth:agar MIC ratios for ceftibuten alone and ceftibuten/avibactam

Species	Isolate No.	Beta-Lactamase & Antibiotic-Resistance Gene Content	CTB (Broth MIC)	CTB (Agar MIC)	CTB Broth:Agar MIC ratio	CTB/AVI (Broth MIC)	CTB/AVI (Agar MIC)	CTB/AVI Broth:Agar MIC ratio
E. coli	ATCC 25922	QC; non-ESBL	0.25	0.25	1	0.12/4	0.06/4	2
E. coli	NCTC 13353	QC; CTX-M-15	32	16	2	0.12/4	0.06/4	2
E. coli	CDC 0369	CTX-M-15	16	4	4	0.03/4	0.03/4	1
E. coli	NCTC 13353	QC; CTX-M-15	16	16	1	0.06/4	0.06/4	1
E. coli	CDC 0451	KPC	4	4	1	0.12/4	0.12/4	1
E. coli	CDC 0452	NDM	>64	>64	NA	>64/4	>64/4	NA
E. coli	CDC 0435	NDM-1	>64	>64	NA	>64/4	>64/4	NA
E. coli	CDC 0503	CTX-M-15; NDM-1; OXA-181	>64	>64	NA	>64/4	64/4	NA
E. coli	ATCC BAA-2523	OXA-48	4	2	2	0.03/4	0.03/4	1
E. coli	ATCC 35218	TEM-1	0.12	0.12	1	0.03/4	0.015/4	2
E. coli	MMX 2234	DHA; SHV; TEM	64	32	2	1/4	0.12/4	8
E. coli	MMX 2269	CTX-M-1; TEM	8	2	4	0.06/4	0.06/4	1
E. coli	MMX 2504	CTX-M-1; OXA-1; SHV	16	16	1	0.25/4	0.12/4	2
E. coli	MMX 5391	CTX-M-1	8	8	1	0.06/4	0.06/4	1
E. coli	MMX 5741	CTX-M-1; OXA-1; OXA-9; TEM	16	4	4	0.06/4	0.06/4	1
E. coli	MMX 5743	CTX-M-1; OXA-1; TEM	64	32	2	0.25/4	0.25/4	1
E. coli	MMX 5744	CTX-M-1; OXA-1	64	32	2	0.12/4	0.12/4	1
E. coli	MMX 5745	KPC; TEM	8	8	1	0.12/4	0.25/4	0.5
E. coli	MMX 5746	CTX-M-1; OXA-1; SHV	8	8	1	0.06/4	0.12/4	0.5

Species	Isolate No.	Beta-Lactamase & Antibiotic-Resistance Gene Content	CTB (Broth MIC)	CTB (Agar MIC)	CTB Broth:Agar MIC ratio	CTB/AVI (Broth MIC)	CTB/AVI (Agar MIC)	CTB/AVI Broth:Agar MIC ratio
E. coli	MMX 2506	CTX-M-1; OXA-1; TEM	32	8	4	0.12/4	0.06/4	2
E. coli	MMX 5749	CTX-M-1; OXA-1; TEM	16	32	0.5	0.06/4	0.06/4	1
E. coli	MMX 5751	SHV; TEM	64	8	8	0.06/4	0.06/4	1
E. coli	MMX 5755	OXA-1; SHV; TEM	32	16	2	0.12/4	0.12/4	1
E. coli	MMX 5756	OXA-9; SHV; TEM	8	2	4	0.12/4	0.12/4	1
E. coli	MMX 5758	CTX-M-1; SHV; TEM	8	4	2	0.06/4	0.03/4	2
E. coli	MMX 5759	CMY-2; CMY-41; OXA-1; OXA-9; SHV; TEM	>64	>64	NA	4/4	1/4	4
E. coli	MMX 5762	OXA-9; SHV; TEM	2	2	1	0.12/4	0.12/4	1
E. coli	MMX 2513	SVH; TEM	8	4	2	0.5/4	0.25/4	2
E. coli	MMX 5768	CTX-M-1; OXA-1; SHV; TEM	16	8	2	0.06/4	0.06/4	1
E. coli	MMX 5772	CTX-M-1; OXA-1	16	8	2	0.12/4	0.06/4	2
E. coli	MMX 1391	Non-ESBL	0.12	0.06	2	0.03/4	0.015/4	2
E. coli	MMX 2214	Non-ESBL	0.5	0.5	1	0.06/4	0.06/4	1
E. coli	MMX 5300	Non-ESBL	0.03	0.03	1	0.015/4	0.015/4	1
E. coli	MMX 2246	Non-ESBL	0.5	0.5	1	0.06/4	0.03/4	2
E. coli	MMX 2247	Non-ESBL	0.25	0.25	1	0.03/4	0.03/4	1

Species	Isolate No.	Beta-Lactamase & Antibiotic-Resistance Gene Content	CTB (Broth MIC)	CTB (Agar MIC)	CTB Broth:Agar MIC ratio	CTB/AVI (Broth MIC)	CTB/AVI (Agar MIC)	CTB/AVI Broth:Agar MIC ratio
E. coli	CDC0048	aadA2, aph(3')-Ia, catA1, CMY-6, CTX-M-15, dfrA12, dfrA29, NDM-1, OmpF, OXA-2, rmtC, strA, strB, sul1, TEM-1B, tet(B)	>64	>64	NA	>64/4	>64/4	NA
E. coli	CDC0061	aac(6')-Ib, aadA1, aadA2, dfrA12, dfrA14, KPC-3, OXA-9, strA, strB, sul1, sul2, sul3, TEM-1A, tet(A)	2	1	2	0.03/4	0.03/4	1
K. pneumoniae	ATCC BAA-1705	QC; KPC-2, TEM, SHV	8	8	1	0.06/4	0.06/4	1
K. pneumoniae	CDC 0522	KPC-2	32	16	2	0.12/4	0.06/4	2
K. pneumoniae	NCTC 13440	VIM-1	64	64	1	64/4	32/4	2
K. pneumoniae	ATCC 700603	SHV-18; OXA-2; OmpK	1	0.5	2	0.12/4	0.06/4	2
K. pneumoniae	BAA-1705	QC; KPC-2; SHV; TEM	4	4	1	0.06/4	0.12/4	0.5
K. pneumoniae	CDC 0504	SHV-OSBL(b); CTX-M-15; OXA-48	4	8	0.5	0.12/4	0.12/4	1

Species	Isolate No.	Beta-Lactamase & Antibiotic-Resistance Gene Content	CTB (Broth MIC)	CTB (Agar MIC)	CTB Broth:Agar MIC ratio	CTB/AVI (Broth MIC)	CTB/AVI (Agar MIC)	CTB/AVI Broth:Agar MIC ratio
K. pneumoniae	CDC 0523	KPC-2	8	8	1	0.06/4	0.06/4	1
K. pneumoniae	CDC 0524	SHV-12; KPC-3	16	8	2	0.25/4	0.12/4	2
K. pneumoniae	CDC 0525	KPC-11	32	16	2	0.12/4	0.12/4	1
K. pneumoniae	CDC 0438	KPC	32	16	2	0.25/4	0.25/4	1
K. pneumoniae	CDC 0453	KPC	64	64	1	0.5/4	1/4	0.5
K. pneumoniae	CDC 0454	KPC	8	8	1	0.06/4	0.25/4	0.25
K. pneumoniae	CDC 0361	KPC	8	8	1	0.03/4	0.06/4	0.5
K. pneumoniae	CDC 0362	KPC	32	8	4	0.5/4	0.12/4	4
K. pneumoniae	CDC 0363	KPC	8	4	2	0.12/4	0.06/4	2
K. pneumoniae	CDC 0364	KPC	8	8	1	0.06/4	0.12/4	0.5
K. pneumoniae	CDC 0505	SHV-12(e); TEM-OSBL(b); CTX-M-15; NDM-1	>64	>64	NA	>64/4	>64/4	NA
K. pneumoniae	CDC 0506	SHV-ESBL(u); TEM-OSBL(b); CTX-M-15; CTX-M-14; NDM-1	>64	>64	NA	>64/4	>64/4	NA
K. pneumoniae	CDC 0507	SHV-OSBL(b); TEM-OSBL(b); CTX-M-15; NDM-1; OXA-232	>64	>64	NA	>64/4	>64/4	NA

Species	Isolate No.	Beta-Lactamase & Antibiotic-Resistance Gene Content	CTB (Broth MIC)	CTB (Agar MIC)	CTB Broth:Agar MIC ratio	CTB/AVI (Broth MIC)	CTB/AVI (Agar MIC)	CTB/AVI Broth:Agar MIC ratio
K. pneumoniae	MMX 8706	KPC, SHV, TEM	8	8	1	0.12/4	0.06/4	2
K. pneumoniae	MMX 8709	CTX-M-1, OXA-1, SHV, TEM	4	4	1	0.015/4	0.03/4	0.5
K. pneumoniae	MMX 8949	CTX-M-1, OXA-1, SHV, TEM	2	4	0.5	0.03/4	0.03/4	1
K. pneumoniae	MMX 9024	CTX-M-1, OXA-1, SHV	16	16	1	0.03/4	0.03/4	1
K. pneumoniae	MMX 9027	CTX-M-1, OXA-1, SHV, TEM	32	16	2	0.03/4	0.03/4	1
K. pneumoniae	MMX 9029	CTX-M-1, SHV, TEM	16	16	1	0.12/4	0.12/4	1
K. pneumoniae	MMX 9031	CTX-M-1, OXA-1, SHV, TEM	16	8	2	0.03/4	0.03/4	1
K. pneumoniae	MMX 9059	CTX-M-1, SHV	>64	64	NA	0.25/4	0.25/4	1
K. pneumoniae	MMX 9060	CTX-M-1, KPC, SHV	32	8	4	0.03/4	0.06/4	0.5
K. pneumoniae	MMX 9061	CTX-M-1, KPC, SHV, TEM	8	8	1	0.06/4	0.06/4	1
K. pneumoniae	MMX 9062	CTX-M-1, OXA-1, SHV, TEM	16	8	2	0.12/4	0.12/4	1
K. pneumoniae	MMX 9169	SHV, TEM	2	1	2	0.06/4	0.03/4	2
K. pneumoniae	MMX 9033	Non-ESBL	0.12	0.06	2	0.06/4	0.06/4	1
K. pneumoniae	MMX 2238	Non-ESBL	0.12	0.06	2	0.03/4	0.03/4	1

Species	Isolate No.	Beta-Lactamase & Antibiotic-Resistance Gene Content	CTB (Broth MIC)	CTB (Agar MIC)	CTB Broth:Agar MIC ratio	CTB/AVI (Broth MIC)	CTB/AVI (Agar MIC)	CTB/AVI Broth:Agar MIC ratio
K. pneumoniae	MMX 4382	Non-ESBL	2	2	1	2/4	2/4	1
K. pneumoniae	MMX 1349	Non-ESBL	0.03	0.03	1	0.03/4	0.03/4	1
K. pneumoniae	MMX 2548	Non-ESBL	0.015	0.06	0.25	0.015/4	0.015/4	1
K. oxytoca	CDC 0375	SHV-5	2	1	2	0.06/4	0.06/4	1
E. cloacae	CDC 0366	KPC	8	8	1	0.25/4	0.25/4	1
E. cloacae	CDC 0501	VIM-1	64	>64	NA	64/4	>64/4	NA
E. cloacae	CDC 0365	KPC	16	16	1	0.5/4	0.5/4	1
E. cloacae	CDC 0502	SHV-12(e); TEM-OSBL(b); ACT-TYPE; IMP-8	>64	>64	NA	>64/4	>64/4	NA
E. cloacae	CDC 0448	NDM	>64	>64	NA	>64/4	>64/4	NA
E. cloacae	ATCC BAA-1143	ampC	>64	>64	NA	2/4	1/4	2
E. cloacae	CDC0038	-	>64	>64	NA	>64/4	>64/4	NA
E. cloacae	CDC0050	-	32	16	2	0.25/4	0.06/4	4
E. cloacae	CDC0053	-	8	8	1	0.25/4	0.25/4	1
E. cloacae	MMX 4422	-	16	2	8	0.25/4	0.12/4	2
E. cloacae	MMX 4423	-	1	0.5	2	0.03/4	0.03/4	1
E. cloacae	MMX 4424	-	4	4	1	0.03/4	0.03/4	1
E. cloacae	MMX 4425	-	8	8	1	0.5/4	0.25/4	2
E. cloacae	MMX 10684	-	64	32	2	0.25/4	0.12/4	2
E. cloacae	MMX 10685	-	0.5	0.25	2	0.06/4	0.03/4	2
E. cloacae	MMX 10686	-	0.5	2	0.25	0.12/4	0.25/4	0.5
E. cloacae	MMX 10687	-	1	0.5	2	0.06/4	0.03/4	2
E. cloacae	MMX 10688	-	1	0.5	2	0.06/4	0.06/4	1

Species	Isolate No.	Beta-Lactamase & Antibiotic-Resistance Gene Content	CTB (Broth MIC)	CTB (Agar MIC)	CTB Broth:Agar MIC ratio	CTB/AVI (Broth MIC)	CTB/AVI (Agar MIC)	CTB/AVI Broth:Agar MIC ratio
E. cloacae	MMX 10689	-	2	1	2	0.12/4	0.06/4	2
E. cloacae	MMX 10690	-	1	1	1	0.06/4	0.06/4	1
C. rodentium	ATCC 51459	-	0.03	0.06	0.5	0.015/4	0.015/4	1
C. freundii	CDC 0116	aac(6')-If, aadA1, aadB, CMY-76, CMY-79, dfrA1, KPC-2, OmpF, sul1, sul2	>64	>64	NA	1/4	0.5/4	2
C. freundii	CDC 0157	aac(6')-Ib, aadA1, aadA2, armA, catA1, CTX-M-15, dfrA12, erm(B), mph(E), msr(E), NDM-1, OmpF, OXA-9, QnrB34, rmtC, TEM-1B	>64	>64	NA	>64/4	>64/4	NA
C. koseri	MMX 6071	-	0.03	0.03	1	0.015/4	0.015/4	1
C. koseri	MMX 6072	-	0.06	0.06	1	0.03/4	0.06/4	0.5
C. freundii	MMX 6073	-	>64	>64	NA	0.25/4	0.25/4	1
C. freundii	MMX 6074	-	2	2	1	0.06/4	0.06/4	1
C. freundii	MMX 6075	-	2	1	2	0.06/4	0.03/4	2
C. freundii	MMX 6076	-	1	1	1	0.03/4	0.06/4	0.5
C. freundii	MMX 6077	-	2	1	2	0.06/4	0.06/4	1
C. freundii	MMX 6078	-	>64	64	NA	0.06/4	0.06/4	1
C. freundii	MMX 6225	-	1	1	1	0.06/4	0.03/4	2
C. freundii	MMX 6226	-	2	2	1	0.12/4	0.03/4	4
C. freundii	MMX 6227	-	1	2	0.5	0.12/4	0.06/4	2
C. freundii	MMX 6228	-	2	1	2	0.03/4	0.03/4	1

Species	Isolate No.	Beta-Lactamase & Antibiotic-Resistance Gene Content	CTB (Broth MIC)	CTB (Agar MIC)	CTB Broth:Agar MIC ratio	CTB/AVI (Broth MIC)	CTB/AVI (Agar MIC)	CTB/AVI Broth:Agar MIC ratio
C. freundii	MMX 6284	-	8	4	2	0.06/4	0.06/4	1
C. koseri	MMX 6360	-	0.06	0.25	0.25	0.03/4	0.06/4	0.5
C. koseri	MMX 6361	-	0.03	0.06	0.5	0.03/4	0.03/4	1
C. koseri	MMX 6362	-	0.06	0.03	2	0.06/4	0.03/4	2
C. koseri	MMX 6363	-	0.12	0.03	4	0.03/4	0.03/4	1
P. mirabilis	CDC 0155	aadA1, aadA5, dfrA1, dfrA17, KPC-6, sul1, sul2, tet(J)	2	0.5	4	0.03/4	0.015/4	2
P. mirabilis	CDC 0156	cmlA1, dfrA1, dfrA14, KPC-2, OXA-10, sul1, sul2, tet(D), tet(J)	0.06	0.015	4	0.25/4	0.015/4	16
P. mirabilis	CDC 0159	aadA2, armA, dfrA1, dfrA12, mph(E), msr(E), NDM-1, strA, strB, sul1, sul2	64	32	2	64/4	64/4	1
P. mirabilis	ATCC 29906	-	0.015	0.015	1	0.015/4	0.015/4	1
P. vulgaris	ATCC 33420	-	2	0.03	64	0.03/4	0.015/4	2
P. mirabilis	CDC 0059	aadA1, aadA2, aadB, aph(3')-Ic, dfrA1, sul1, TEM-1B, tet(J)	2	2	1	2/4	1/4	2
P. mirabilis	CDC 0377	KPC-3, TEM-1	0.06	0.06	1	0.015/4	0.008/4	2
P. mirabilis	CDC 0379	TEM-1, VEB-1A	1	0.25	4	0.015/4	0.015/4	1
P. vulgaris	MMX 6453	-	2	0.008	256	0.008/4	0.008/4	1
P. vulgaris	MMX 6454	-	0.06	0.03	2	0.015/4	0.03/4	0.5
P. vulgaris	MMX 8483	-	2	0.03	64	0.03/4	0.015/4	2

Species	Isolate No.	Beta-Lactamase & Antibiotic-Resistance Gene Content	CTB (Broth MIC)	CTB (Agar MIC)	CTB Broth:Agar MIC ratio	CTB/AVI (Broth MIC)	CTB/AVI (Agar MIC)	CTB/AVI Broth:Agar MIC ratio
P. vulgaris	MMX 8484	-	1	0.03	32	0.015/4	0.015/4	1
P. vulgaris	MMX 8485	-	0.03	0.015	2	0.015/4	0.008/4	2
P. mirabilis	MMX 8486	-	0.25	0.25	1	0.06/4	0.06/4	1
P. mirabilis	MMX 8487	-	0.06	0.03	2	0.015/4	0.015/4	1
P. mirabilis	MMX 8488	-	0.015	0.015	1	0.015/4	0.015/4	1
P. penneri	MMX 8489	-	0.03	0.03	1	0.015/4	0.015/4	1
P. mirabilis	MMX 8490	-	0.06	0.015	4	0.03/4	0.015/4	2
P. mirabilis	MMX 8491	-	>64	>64	NA	>64/4	>64/4	NA
P. mirabilis	MMX 8492	-	0.03	0.03	1	0.015/4	0.03/4	0.5
S. marcescens	CDC 0520	FOX-5	16	16	1	0.5/4	0.25/4	2
S. marcescens	CDC 0521	CMY-16	>64	>64	NA	4/4	4/4	1
S. marcescens	MMX 9155	-	0.25	0.25	1	0.12/4	0.12/4	1
S. marcescens	MMX 9272	-	0.25	0.25	1	0.12/4	0.12/4	1
M. morganii	CDC 0519	DHA-TYPE	>64	>64	NA	0.5/4	0.5/4	1
M. morganii	MMX 6234	-	0.5	0.25	2	0.03/4	0.015/4	2
M. morganii	MMX 6429	-	1	0.25	4	0.03/4	0.015/4	2
M. morganii	MMX 6431	-	0.5	0.12	4	0.015/4	0.015/4	1
M. morganii	MMX 6573	-	0.5	0.12	4	0.03/4	0.015/4	2
M. morganii	MMX 6575	-	16	32	0.5	0.12/4	0.12/4	1
M. morganii	MMX 6609	-	4	2	2	0.015/4	0.015/4	1
M. morganii	MMX 6611	-	32	2	16	16/4	2/4	8
M. morganii	MMX 6613	-	2	2	1	0.015/4	0.015/4	1
P. rettgeri	MMX 8504	-	0.03	0.03	1	0.015/4	0.015/4	1
P. rettgeri	MMX 8596	-	0.015	0.004	4	0.015/4	0.004/4	4

Species	Isolate No.	Beta-Lactamase & Antibiotic-Resistance Gene Content	CTB (Broth MIC)	CTB (Agar MIC)	CTB Broth:Agar MIC ratio	CTB/AVI (Broth MIC)	CTB/AVI (Agar MIC)	CTB/AVI Broth:Agar MIC ratio
P. rettgeri	MMX 8602	-	0.008	0.004	2	0.008/4	0.004/4	2
P. rettgeri	MMX 8605	-	0.008	0.004	2	0.004/4	0.008/4	0.5
P. stuartii	MMX 8511	-	0.008	0.008	1	0.004/4	0.008/4	0.5
P. stuartii	MMX 8611	-	0.03	0.06	0.5	0.03/4	0.015/4	2

CTB, ceftibuten; AVI, avibactam; NA, not applicable.

Table S3. Line listing of broth and agar MIC values (μg/mL) for ceftibuten/avibactam against 20 replicates of QC isolates across 2 run days

Day	Replicate	CTB/AVI MIC (μg/mL)							
		E. coli ATCC 25922		*E. coli* NCTC 13353		*K. pneumoniae* ATCC BAA-1705		*K. pneumoniae* ATCC 700603	
		BMD	AD	BMD	AD	BMD	AD	BMD	AD
1	1	0.06/4	0.03/4	0.12/4	0.06/4	0.12/4	0.25/4	0.12/4	0.25/4
	2	0.06/4	0.03/4	0.12/4	0.06/4	0.25/4	0.25/4	0.12/4	0.25/4
	3	0.06/4	0.03/4	0.12/4	0.06/4	0.12/4	0.25/4	0.12/4	0.25/4
	4	0.06/4	0.03/4	0.12/4	0.03/4	0.12/4	0.25/4	0.12/4	0.25/4
	5	0.06/4	0.03/4	0.12/4	0.06/4	0.12/4	0.25/4	0.12/4	0.25/4
	6	0.06/4	0.03/4	0.12/4	0.06/4	0.12/4	0.25/4	0.12/4	0.25/4
	7	0.06/4	0.03/4	0.12/4	0.06/4	0.12/4	0.25/4	0.12/4	0.25/4
	8	0.12/4	0.03/4	0.12/4	0.06/4	0.12/4	0.25/4	0.25/4	0.25/4
	9	0.06/4	0.06/4	0.06/4	0.06/4	0.12/4	0.25/4	0.12/4	0.25/4
	10	0.12/4	0.06/4	0.06/4	0.06/4	0.12/4	0.25/4	0.12/4	0.25/4
2	11	0.12/4	0.03/4	0.12/4	0.03/4	0.12/4	0.12/4	0.25/4	0.25/4
	12	0.12/4	0.03/4	0.12/4	0.03/4	0.12/4	0.12/4	0.12/4	0.25/4
	13	0.06/4	0.03/4	0.12/4	0.06/4	0.12/4	0.12/4	0.12/4	0.25/4
	14	0.06/4	0.03/4	0.12/4	0.06/4	0.25/4	0.12/4	0.12/4	0.25/4
	15	0.06/4	0.03/4	0.12/4	0.06/4	0.25/4	0.12/4	0.12/4	0.25/4
	16	0.12/4	0.03/4	0.5/4	0.03/4	0.12/4	0.12/4	0.12/4	0.25/4
	17	0.12/4	0.03/4	0.12/4	0.06/4	0.12/4	0.12/4	0.12/4	0.25/4
	18	0.06/4	0.03/4	0.06/4	0.06/4	0.12/4	0.12/4	0.25/4	0.25/4
	19	0.06/4	0.03/4	0.12/4	0.06/4	0.12/4	0.12/4	0.25/4	0.25/4
	20	0.06/4	0.03/4	0.12/4	0.06/4	0.12/4	0.12/4	0.12/4	0.25/4
QC Range:		0.016/4-0.12/4	NA	0.03/4-0.12/4	NA	0.03/4-0.25/4	NA	0.06/4-0.25/4	NA

CTB/AVI, ceftibuten/avibactam; BMD, broth microdilution; AD, agar dilution.
